# Transcriptomic insights into the effects of tyrosine on sub-Columbian plumage in H line chickens

**DOI:** 10.3389/fvets.2025.1720520

**Published:** 2025-11-28

**Authors:** Xinlei Wang, Liheng Zhang, Liyu Yang, Pengkun Yang, Yingying Qiao, Zhanbing Han, Jiaying Fan, Qiang Li, Dingding Zhang, Zhuanjian Li, Xiangtao Kang, Juan Du, Ruiting Li

**Affiliations:** 1 College of Animal Science and Technology, Henan University of Animal Husbandry and Economy, Zhengzhou, China; 2 Henan Key Laboratory of Healthy Breeding and Efficient Reproduction of Livestock and Poultry, Zhengzhou, China; 3 College of Animal Science and Technology, Henan Agricultural University, Zhengzhou, China; 4 Key Research Project of the Shennong Laboratory, Zhengzhou, China; 5 Zhengzhou Health College, Zhengzhou, China

**Keywords:** chicken, sub-Columbian plumage, *EDNRB2*, tyrosine, RNA-seq

## Abstract

Tyrosine is known to influence melanin generation; however, its involvement in melanin production in chicken feathers is unknown. We evaluated the feather color of H-line chickens fed diets containing different concentrations of tyrosine (0, 0.4, 0.6, 0.8, and 1.0%). The results indicated that a diet containing 1.0% tyrosine fed for 40 days significantly increased melanin deposition in the feathers (*p* < 0.05). Following this observation, we collected feather follicle tissue from chickens fed either 0% or 1.0% tyrosine at the 40-day time point for transcriptome sequencing. RNA-seq analysis identified a total of 314 DEGs, comprising 116 upregulated and 198 downregulated genes. KEGG analysis of feather follicle tissue revealed that 7 DEGs (*EDNRB2, WNT3, POMC, INS, FLT3, CACNA2D3*, and *CACNA1I*) mapped to melanin-related pathways, including the melanogenesis, MAPK signaling and Wnt signaling pathways. We also identified specific protein interactions within the melanin pathway, including EDNRB2–MLPH and WNT3–FGF16 interactions. Notably, the expression level of the *EDNRB2* gene reached its peak at 10 weeks within the 0–12 week growth period in H-line chickens. In primary chicken melanocytes, *EDNRB2* expression was quantified following tyrosine supplementation and was found to be markedly elevated at a concentration of 10^−6^ mol/L, significantly higher than the control and other treatment groups (*p* < 0.05). Overall, our findings suggest the significant involvement of the tyrosine-induced *EDNRB2* regulatory network in melanin levels in sub-Columbian plumage. Taken together, these findings increase our understanding of the molecular mechanisms that regulate tyrosine-mediated melanin deposition in chicken plumage.

## 1 Introduction

The chicken, with diverse plumage color combinations and patterns, ranks among the most colorful terrestrial vertebrates, thereby capturing the interest of researchers (1). Feather color not only attracts the opposite sex but also deters predators from chickens. Feather color is an important external characteristic in chickens (2). It not only is a significant identifying feature for different breeds but also influences consumers’ choices when selecting high-quality live poultry in markets, so it holds economic significance (3). With the rapid development of integrated agriculture, improving feed nutrition to maintain and improve feather color is essential. Altering the plumage color phenotype by adding nutrients to the feed, with the aim of identifying key genes that influence this phenotype, has become an important approach in poultry breeding.

Chicken feather color is determined by the quantity and distribution of melanin, which are dependent on the interaction between genotype and nutrition (4). Modern technological advances and scientific research have resulted in the identification of numerous mutations in genes affecting chicken feather color, including dominant white (I), recessive white (c), barring (B), spots (mo), extended black (E), dark brown (DB), and Columbia, among others (5–9). Nutritional factors can also affect melanin deposition in animals (10). For example, a combination of vitamin C and vitamin E supplementation can be used to treat age spots or melasma in humans (11). Additional studies have demonstrated a notable decrease in UV-induced skin redness and enhancements in skin hydration and elasticity after the oral administration of lutein (12). Similarly, goldfish fed spirulina and astaxanthin-enriched brine shrimp presented improvements in pigmentation and skin redness indices (13). However, there have been no reports on the impact of exogenous nutrient supplementation on chicken feather color.

Melanin synthesis is performed by melanocytes in melanosomes (14), which then transfer melanin granules to adjacent keratinocytes, resulting in melanin accumulation and, in chickens, the production of pigmented feathers (15). In mature melanocytes, tyrosine is acted upon by tyrosinase and tyrosinase-related proteins to produce DOPA, which subsequently undergoes further oxidation to dopaquinone. Finally, after further reactions, dopaquinone is converted to melanin, which affects fur color, body color, and skin color phenotypes (16, 17). At present, some reports indicate that the addition of moderate amounts of tyrosine can promote the formation of melanin in animals. For example, Li reported that adding tyrosine to the diet can enhance melanin deposition in the breast muscles of Xichuan black-boned chickens (18). A tyrosine-deficient diet can cause black cat fur to become reddish-brown (19). By adjusting the concentrations of copper and specific amino acids, such as phenylalanine and tyrosine, in the diet, the deposition of red pigments in the white fur of dogs can be significantly increased (20). Additionally, increasing the intake of tyrosine in the diet can increase the deposition of black pigments in dog fur (21). However, the effects of a tyrosine diet on melanin deposition in chicken feathers and the underlying mechanism are still unclear.

**Figure 1:**
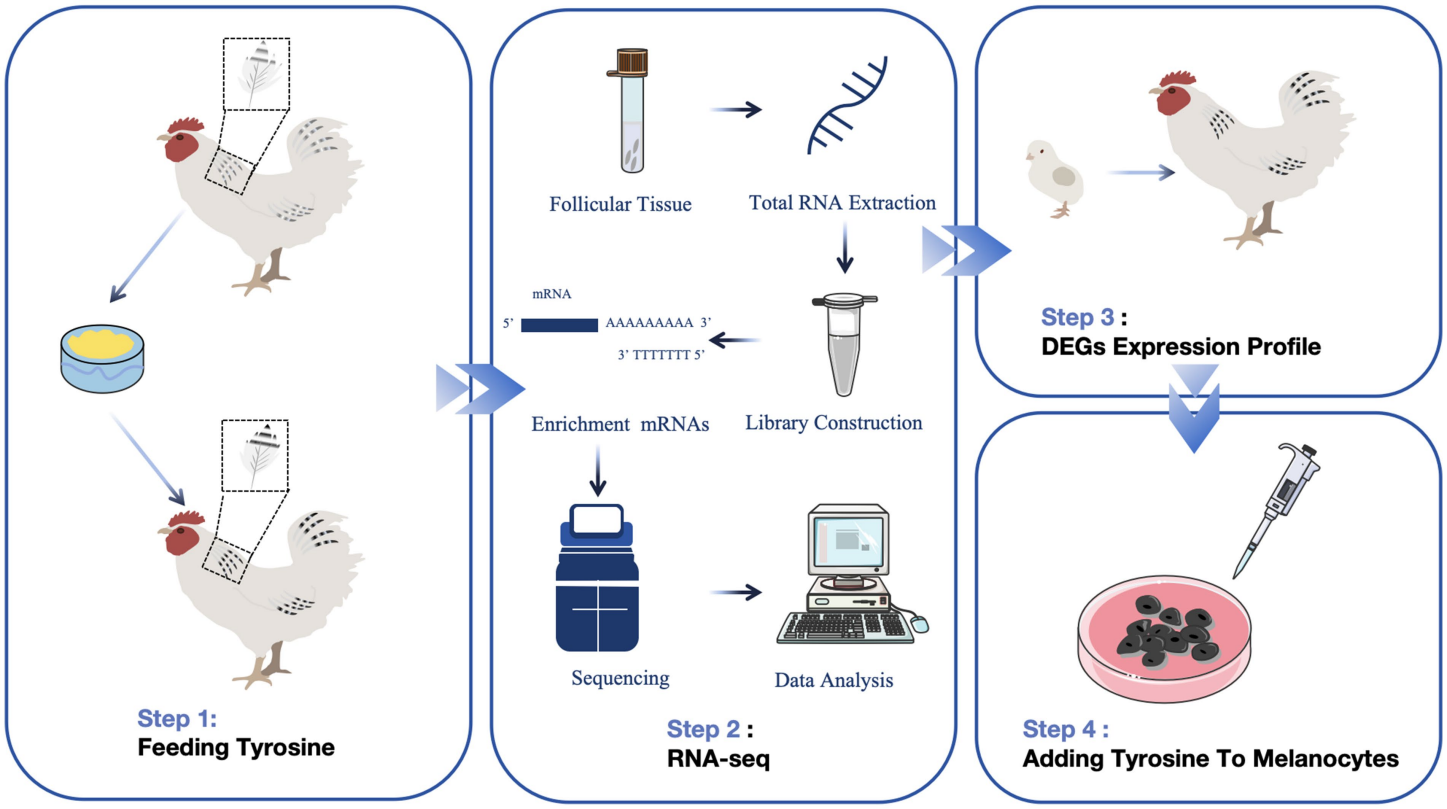
Flow chart for screening and validating the effects of adding tyrosine to the diet on plumage color-associated genes in H line chickens.

Three “Yufen I” hybrid lines were established from Chinese commercial laying hens by Henan Agricultural University. The H line is characterized by mostly white feathers, accompanied by black barring on the primary and secondary feathers, as well as the hackles and tail. While this coloration resembles the Columbian plumage, it involves black barring rather than verticle stripes and is thus termed “sub-Columbian” (22). Theoretical studies on the Columbian plumage pattern began as early as 1955 (23). Previous studies in our laboratory have shown that sub-Columbian plumage is influenced by *SLC45A2* and *CDKN2A*, which are inherited as dominant companions (22). Moreover, transcriptome sequencing of the sub-Columbian feathers, including black and white follicles from the dorsal neck region and white follicles from the abdominal neck, revealed that *MED23* and *GNAQ* are essential for melanin accumulation (24). Additionally, by analyzing transcriptome data from feather follicles from differently colored chickens (yellow, sub-Columbian, and silver) in their necks and wings, it was determined that elevated *SLC45A2* and *GPNMB* levels could enhance melanin deposition in feathers from H-line chickens (25). Although the genetic foundation of feather color in H line chickens is relatively well understood, the regulatory molecular mechanisms underlying melanin deposition in feathers through the addition of tyrosine in feed are not widely known. *EDNRB2*, a gene crucial for melanoblast differentiation and migration along the dorsolateral pathway, plays a significant role in controlling melanin deposition in the muscles of black-boned chickens (18, 26, 27). However, whether the expression of *EDNRB2* is regulated by tyrosine, thereby influencing chicken plumage coloration, remains an unanswered question. This study focuses on H line hens as the research subjects, establishing a no-tyrosine group and groups with varying concentrations of tyrosine in the feed. Samples were collected at different times to determine the time and concentration of tyrosine addition that significantly affect feather color changes. The transcriptomes of neck follicles from untreated controls and tyrosine-treated chickens were sequenced, and the differentially expressed genes (DEGs) affecting feather color changes in follicles under tyrosine supplementation were screened. Using these data, we analyzed the temporal expression characteristics of the candidate gene *EDNRB2* in different stages of H line hens, as well as the expression patterns before and after the addition of tyrosine to melanocytes (Figure 1). This study aimed to determine the optimal tyrosine supplementation in feed to increase melanin deposition in the feather color of H line hens and to elucidate the underlying molecular mechanisms involved.

## 2 Materials and methods

### 2.1 Sample collection

The H line chickens were provided by the Center of Poultry Germplasm Resources of Henan Agricultural University. A total of 200 20-week-old H-line laying hens were randomly assigned to five dietary treatments: one control group and four treatment groups (Groups I-IV). Each group consisted of 4 biological replicates, with 10 hens per replicate. The control group was fed a standard basal diet (Supplementary Table S1) and the four treatment groups received the same basal diet supplemented with tyrosine at concentrations of 0.4, 0.6, 0.8, and 1.0%, respectively. The tyrosine (purity 99%) used for supplementation was obtained from Swire Coca-Cola Drinks Co., Ltd. (Zhengzhou, Henan Province, China). The tyrosine was thoroughly incorporated into the basal diet using a commercial feed mixer to ensure uniform distribution within each formulated feed type. All hens were housed under identical environmental conditions. They were provided with unrestricted access to water and were fed twice daily according to the standard feeding regimen for the laying period.

On the 1st, 12th, 22nd and 40th days after tyrosine addition, the black spots of the nuchal follicle tissue of each group of hens (5 groups of 40) were scored using a YN-15 precision colorimeter (ThreeNH Technology, China), which operates on the CIE L*a*b* color space. Prior to measurement, the instrument was calibrated according to the manufacturer’s protocol using a standard white calibration tile. In the CIE L*a*b* model, the L* value represents lightness on a scale from 0 (pure black) to 100 (pure white), while a* and b* values represent the green-red and blue-yellow axes, respectively. As the plumage in this study exhibited a black-to-white spectrum, only the L* value was adopted for analysis. For each chicken, three feathers from the dorsal neck region were carefully selected. On each feather, a consistent measurement location near the tip was identified. Three replicate measurements were taken at this same spot per feather, ensuring the measuring aperture was in full and perpendicular contact with the feather surface to avoid ambient light interference. This procedure yielded nine raw L* readings per chicken, from which a mean value was calculated and recorded as the definitive neck feather lightness (L*) for the individual.

Based on the analysis results of the L* values, the treatment group with the most significant differences in feather color due to the addition of tyrosine was selected. From this treatment group and the control group, 3 hens were randomly selected from each group (a total of 6) to collect samples of the follicular tissue from their necks. Feather surfaces were wiped with cotton soaked in 75% alcohol, and tweezers were used to clamp the feather root; the follicular tissue was then separated using small forceps and placed in liquid nitrogen.

### 2.2 RNA sequencing

Six follicular tissues from the control (C) and treatment (T) groups were sent to Huada Gene (Shenzhen) for transcriptome sequencing (3 biological replicates in each group). Extraction of total RNA was performed using TRIzol (Invitrogen, United States). A fragment analyzer (Agilent 5200) was used to measure quality parameters such as RNA sample concentration and sample integrity. Six RNA-seq libraries (C1, C2, C3, T1, T2 and T3) were constructed after library purification (1.5% agarose gel electrophoresis). Library sequencing was performed on an Illumina HiSeq 4000 platform. Following the removal of adapter sequences and low-quality data, the raw and high-quality sequence lengths were calculated, along with the Q20 and Q30 values. Cutadapt was used to remove sequences that did not meet the quality criteria to obtain high-quality sequence data, which were then evaluated thoroughly before further analyses were conducted.

### 2.3 Bioinformatics analyses

Clean reads were aligned with the reference genome (Gallus_gallus-5.0) using HISAT (28). Transcript expression levels were determined using RSEM^1^ (29), with quantification as fragments per kilobase million (FPKM) values. EdgeR software (version 3.14.0) was utilized to assess expression variations among the groups to identify DEGs, using the criteria of *p*-value<0.05 and |log_2_FC (fold change)| > 1.5.

GO and KEGG analyses of the DEGs were performed using clusterProfiler in R (v3.6.1), using *p* < 0.05 as the significance threshold for enrichment.

Protein–protein interaction networks (PPI) were constructed to further investigate functional interactions between these DEGs. DEGs were mapped using STRING,^2^ and significant interactions with a combined score of >0.4 were selected for further analysis. Cytoscape (version 3.4.0) was utilized for network visualization.

### 2.4 Spatiotemporal expression pattern analysis

Six healthy one-day-old H line hens were selected from the Henan Agricultural University resource field and raised in individual cages. The neck feather follicle tissues were collected from the animals at 1d, 2, 4, 6, 8, 10, and 12 weeks of age, separated, and frozen in liquid nitrogen for preservation.

### 2.5 Tyrosine treatment of chicken melanocytes

Chicken primary melanocytes were isolated and cultured as described previously (25). Melanocytes were isolated from the peritoneum of Xichuan black-bone chickens at 20 embryonic days. The tissue was washed with PBS containing antibiotics, minced, and digested with dispase II and trypsin–EDTA at 37 °C for 1 h. The digestion was halted by adding supplemented Medium 254. The cell suspension was then filtered through a series of meshes, centrifuged, and the resulting pellet was resuspended to obtain the melanocytes, which were cultured at 37 °C with 5% CO₂. Cells (1 × 10^4^/ml) were grown in normal melanocyte medium, whereas experimental melanocytes were grown in media supplemented with various concentrations of tyrosine (10^−9^, 10^−8^, 10^−7^, and 10^−6^) mol/L for 3 days. The medium was replaced daily with fresh medium containing the corresponding tyrosine concentrations. After the treatment period, cells were collected and stored at −80 °C.

### 2.6 qRT–PCR

RNA was extracted from cells and tissues using TRIzol as above and was reverse-transcribed to cDNA using the Qiagen Reverse Transcription Kit. Primer Premier 6.0 was utilized for primer design; primers were synthesized by Shanghai Sangon Bioengineering Corporation Ltd. (Shanghai, China) and the sequences are given in Supplementary Table S2. The internal reference was *GAPDH*. A series of qRT–PCRs were conducted using 10 ul of SYBR master mix (Vazyme, Nanjing, China), 2 ul of cDNA, 7 ul of ultrapure water, and 0.5 ul of each primer (10 mM). The qRT–PCR program comprised predenaturation for 30 s at 95 °C, 40 3-s cycles at 95 °C and 30 s at 60 °C. The results were calculated as relative expression levels using the 2^−ΔΔCt^ method (30).

### 2.7 Statistical analysis

GraphPad Prism 9 was utilized to calculate, analyze, and plot all the data. Data are expressed as the mean ± standard error of the mean (S. E. M.). One-way ANOVA and Student’s t-test were used for statistical analysis. Data are shown as means ± standard errors with *p* < 0.05 considered statistically significant.

## 3 Results

### 3.1 Feather color analysis after tyrosine feeding to H line chickens

**Figure 2:**
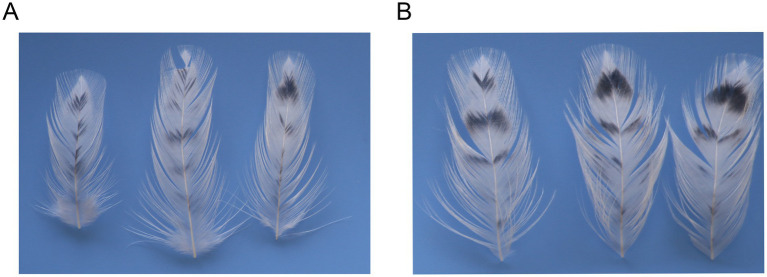
Phenotypic effect of tyrosine on feather color. Representative feathers from the dorsal neck of hens fed a control (0% tyrosine) **(A)** or a 1.0% tyrosine-supplemented **(B)** diet for 40 days.

An experiment in which tyrosine was added to the feed of H line hens revealed that a higher tyrosine concentration in the feed was associated with a lower feather L* value, that is, darker black spots. In addition, a longer duration of tyrosine feeding was correlated with a lower feather color L* value (Table 1). Feather melanin spot L* values were significantly lower in chickens fed a 1.0% tyrosine diet compared to those on a tyrosine-free diet after 40 days (*p* < 0.05) (Figure 2). Furthermore, within the 1.0% tyrosine group, the L* value decreased progressively over time, with the value at 40 days being significantly lower than at the start of the experiment (*p* < 0.05). This study found that feeding H line chickens with a diet containing 1.0% tyrosine for 40 days significantly increased the deposition of melanin in their feathers (*p* < 0.05).

**Table 1:** Effects of dietary tyrosine concentration and supplementation duration on feather melanin spot L* values.

Content	Time			
	40d	40d	40d	40d
0	69.380 ± 7.397	68.110 ± 6.714	64.683 ± 7.526	65.447 ± 6.207^A^
0.4%	64.258 ± 9.188	63.899 ± 9.448	60.198 ± 10.579	58.801 ± 8.835^AB^
0.6%	70.033 ± 9.334^a^	64.581 ± 8.718^ab^	61.232 ± 10.377^b^	58.764 ± 10.688^bAB^
0.8%	66.801 ± 10.682^a^	63.375 ± 11.080^ab^	61.150 ± 12.767^ab^	57.130 ± 12.945^bB^
1.0%	67.438 ± 9.035^a^	61.072 ± 7.268^ab^	56.889 ± 9.197^b^	54.572 ± 5.715^cB^

Values in the table represent the mean L* (lightness) of melanin spots measured by a colorimeter (*n* = 200, 40 per group). L* represents the intensity of brightness; a higher value indicates a less blackness, and vice versa. In the same row, values with different small letter superscripts mean significant difference (*p* < 0.05); While with the same small letter superscripts mean no significant difference (*p* > 0.05). In the same column, values with different uppercase letters superscripts mean significant difference (*p* < 0.05); While with the same uppercase letter superscripts mean no significant difference (*p* > 0.05).

### 3.2 Descriptive statistics of the RNA-seq data

Follicle tissue gene expression in the C1, C2, C3, T1, T2, and T3 groups were analyzed by RNA-seq, and 43.04 ~ 44.07 MB of filtered clean reads were obtained. The per-sample mapping rate against the reference genome was between 82.40 and 86.95%. The average Q20 was 96.47%, and the average Q30 was 87.72% (Supplementary Table S3). Thus, the quality of the data was sufficient for further analysis.

### 3.3 DEG analysis

**Figure 3:**
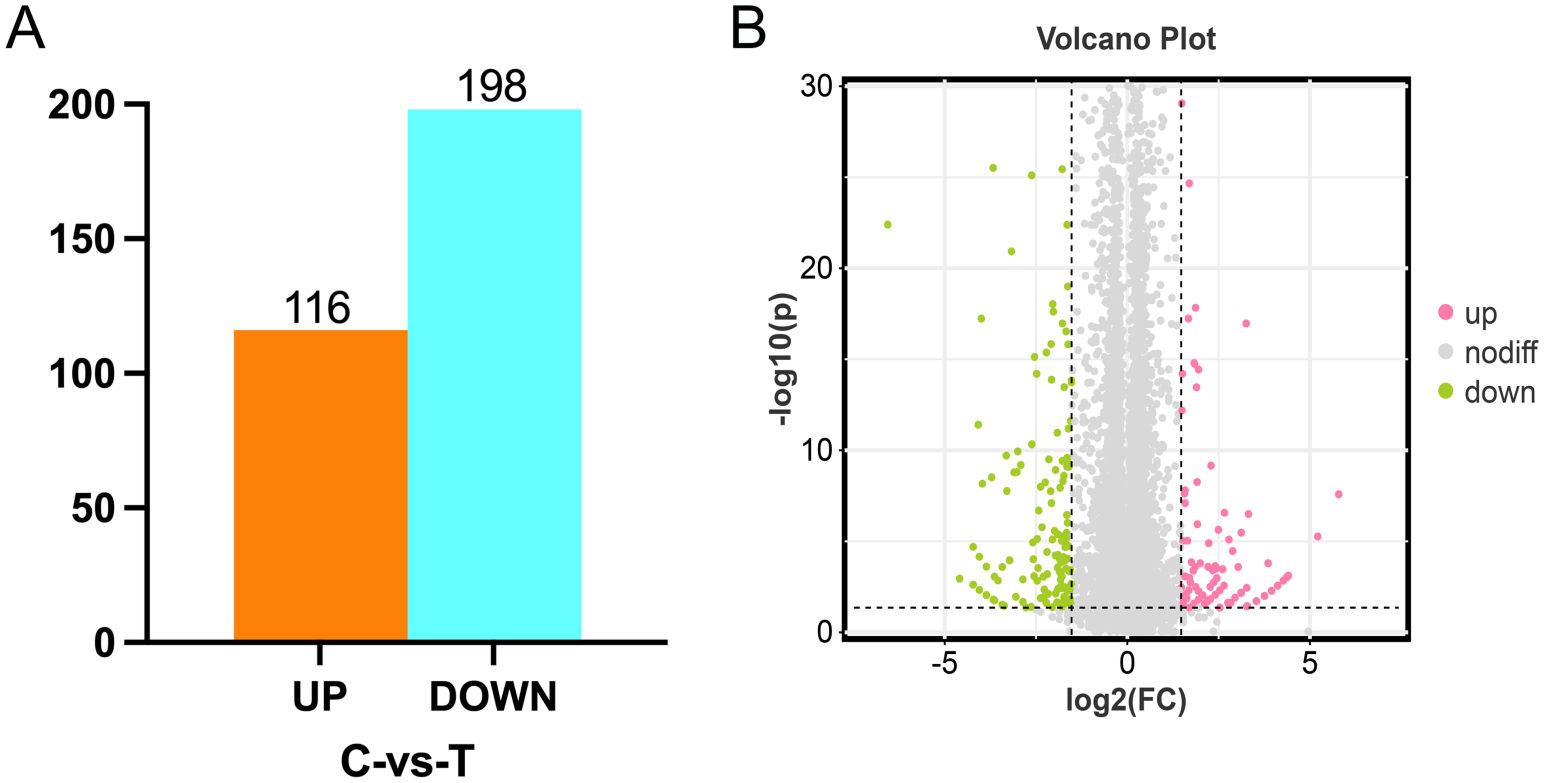
Follicle tissue DEGs between the C and T groups. **(A)** Up- and down-regulated gene numbers. **(B)** Volcano plot of genes identified by RNA-seq, including both upregulated and downregulated genes.

To clarify the pathways underlying the effects of tyrosine on feather color, DEGs between the control and tyrosine-fed follicles were analyzed. Overall, 13,956 genes were detected, from which 314 DEGs were identified between the C and T groups (*p* < 0.05). Of these, 116 were upregulated and 198 were downregulated in the T group (Figure 3). Notably, these DEGs included two marker genes of melanin deposition, *EDNRB2* and *MLPH*.

### 3.4 Verification by qRT-PCR

**Figure 4:**
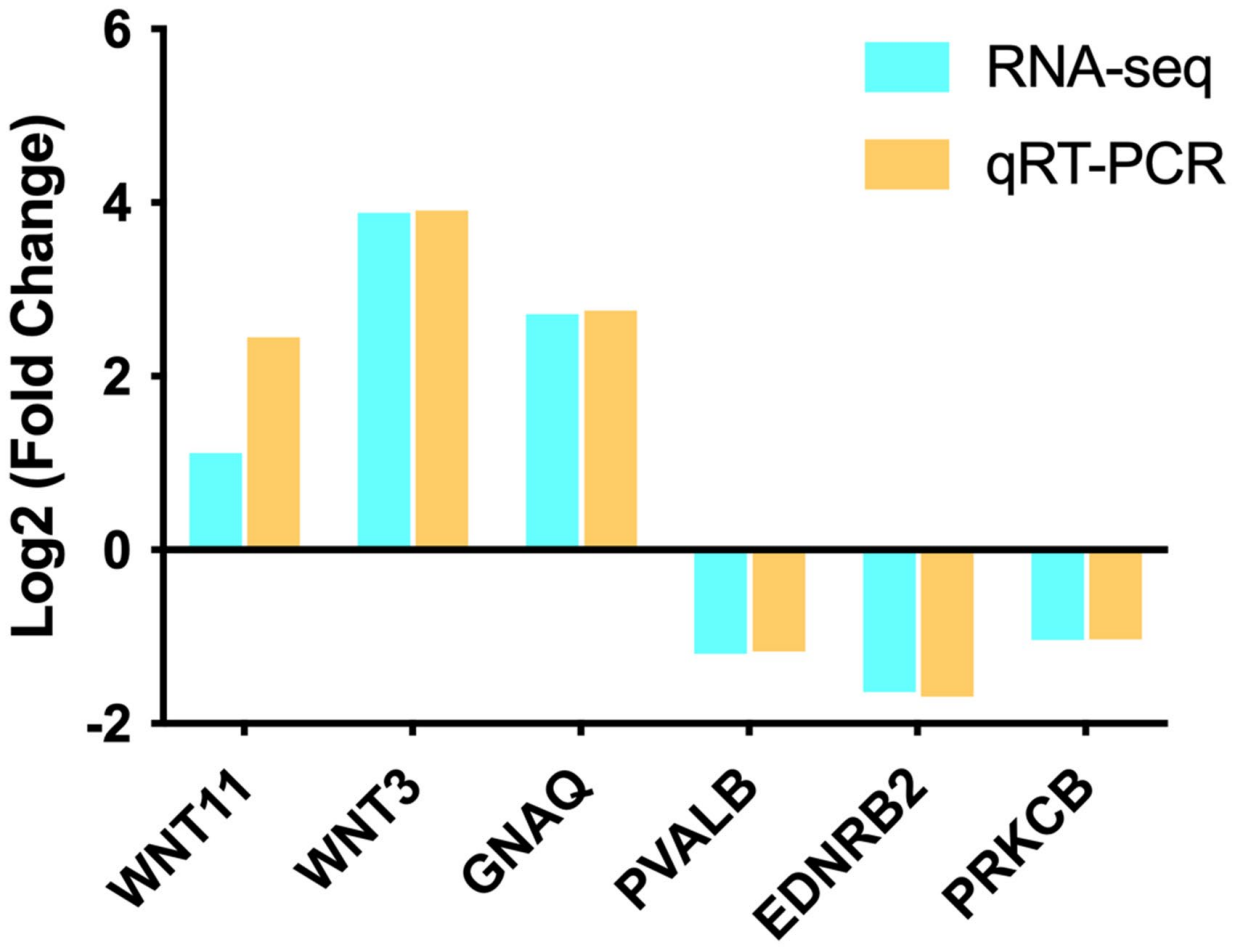
qRT–PCR verification of the RNA-seq data (*n* = 3).

The accuracy and reliability of the RNA-seq data were verified by randomly selecting six genes (*WNT11, WNT3, GNAQ, PVALB, EDNRB2*, and *PRKCB*) for qRT-PCR analysis. Assessment of expression and comparison with the RNA-seq data demonstrated the credibility of the RNA-seq data (Figure 4).

### 3.5 GO analysis

**Figure 5:**
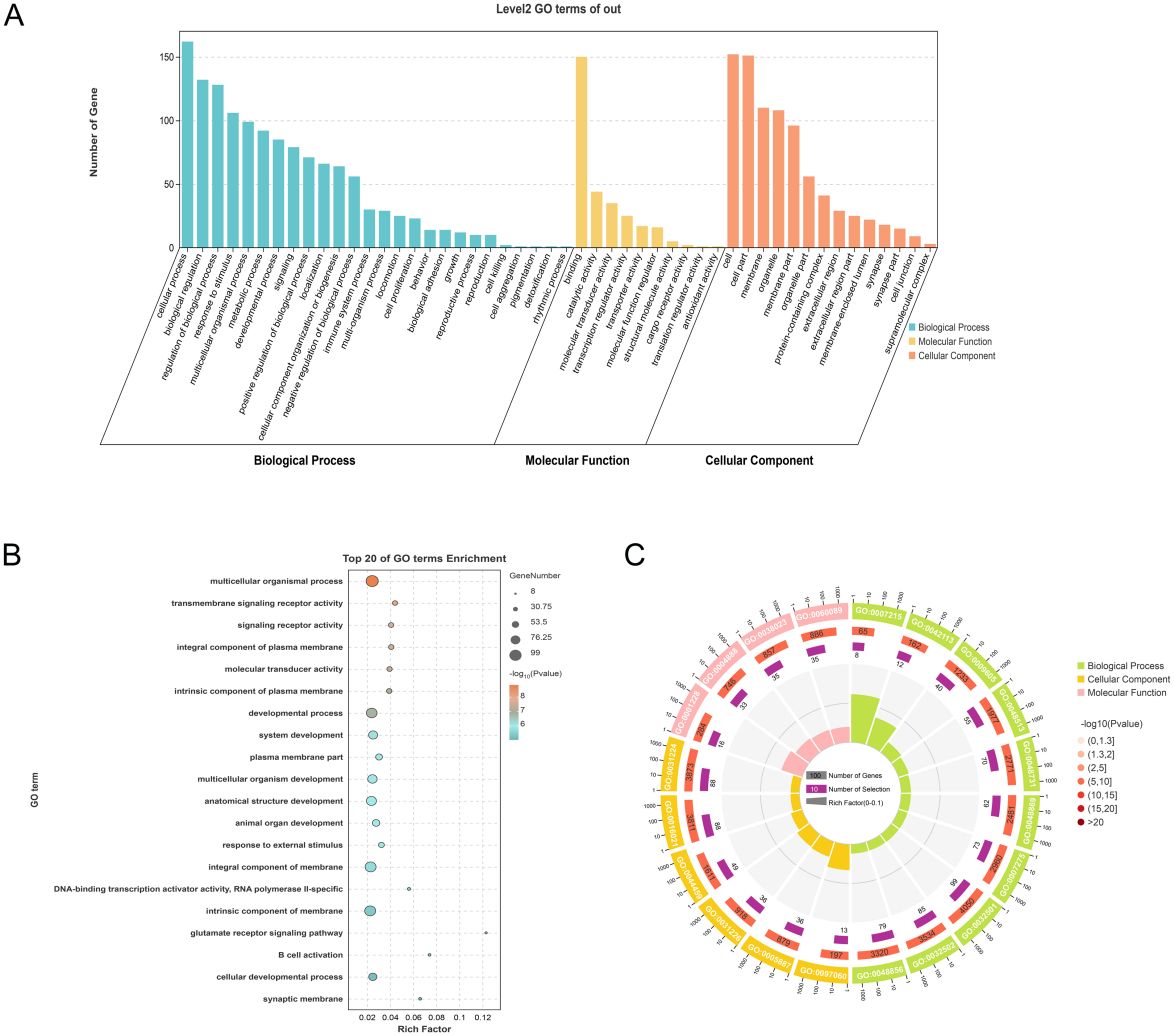
GO enrichment analysis of DEGs. **(A)** DEG GO classifications. **(B)** Bubble diagram showing top 20 enriched GO terms. **(C)** GO enrichment circle.

The functions of the DEGs were analyzed further by GO enrichment. The GO classification system encompasses three primary categories, namely, biological process (BP), cellular component (CC), and molecular function (MF). Enrichment in the BP category primarily involved “cellular process,” “cell,” and “cell part,” while in MF, the most enriched subcategories were “binding,” “protein binding,” and “organic cyclic compound binding,” and in CC, “cell,” “cell part,” and “membrane” (Figure 5A). Notably, the DEGs observed among H line hens fed tyrosine were enriched mainly in BP (cellular process, biological regulation and regulation of biological process). Moreover, the DEGs showed marked enrichment in multicellular biological processes, transmembrane signaling receptor activity, and signaling receptor activity (*p* < 0.05) (Figures 5B,C).

### 3.6 KEGG analysis

**Figure 6:**
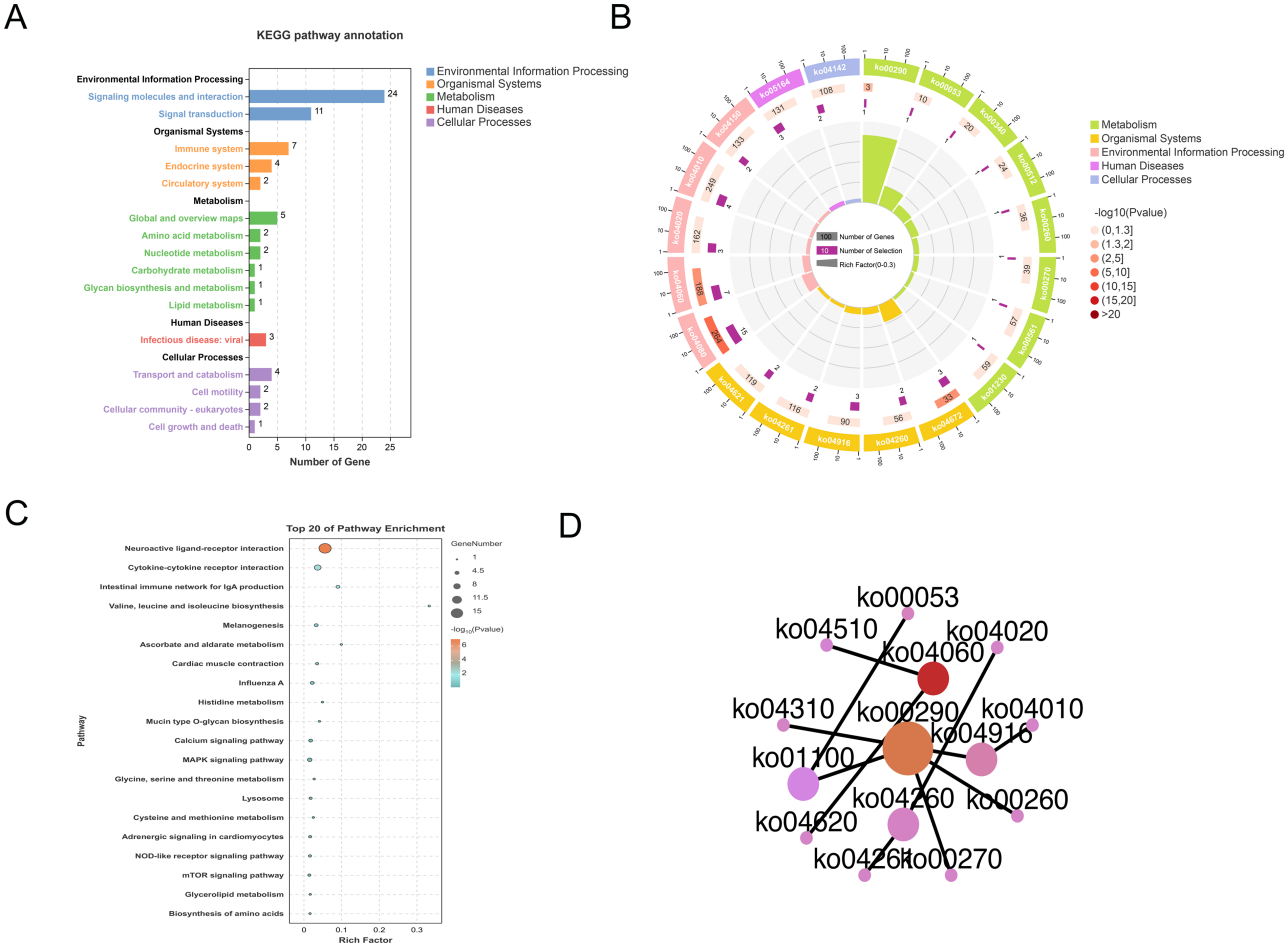
KEGG analysis of DEGs. **(A)** DEG enrichment in KEGG Class A and B pathways. **(B)** KEGG enrichment circle. **(C)** Bubble diagram showing top 20 KEGG pathways. **(D)** Network plot of the interactions between different pathways.

**Figure 7:**
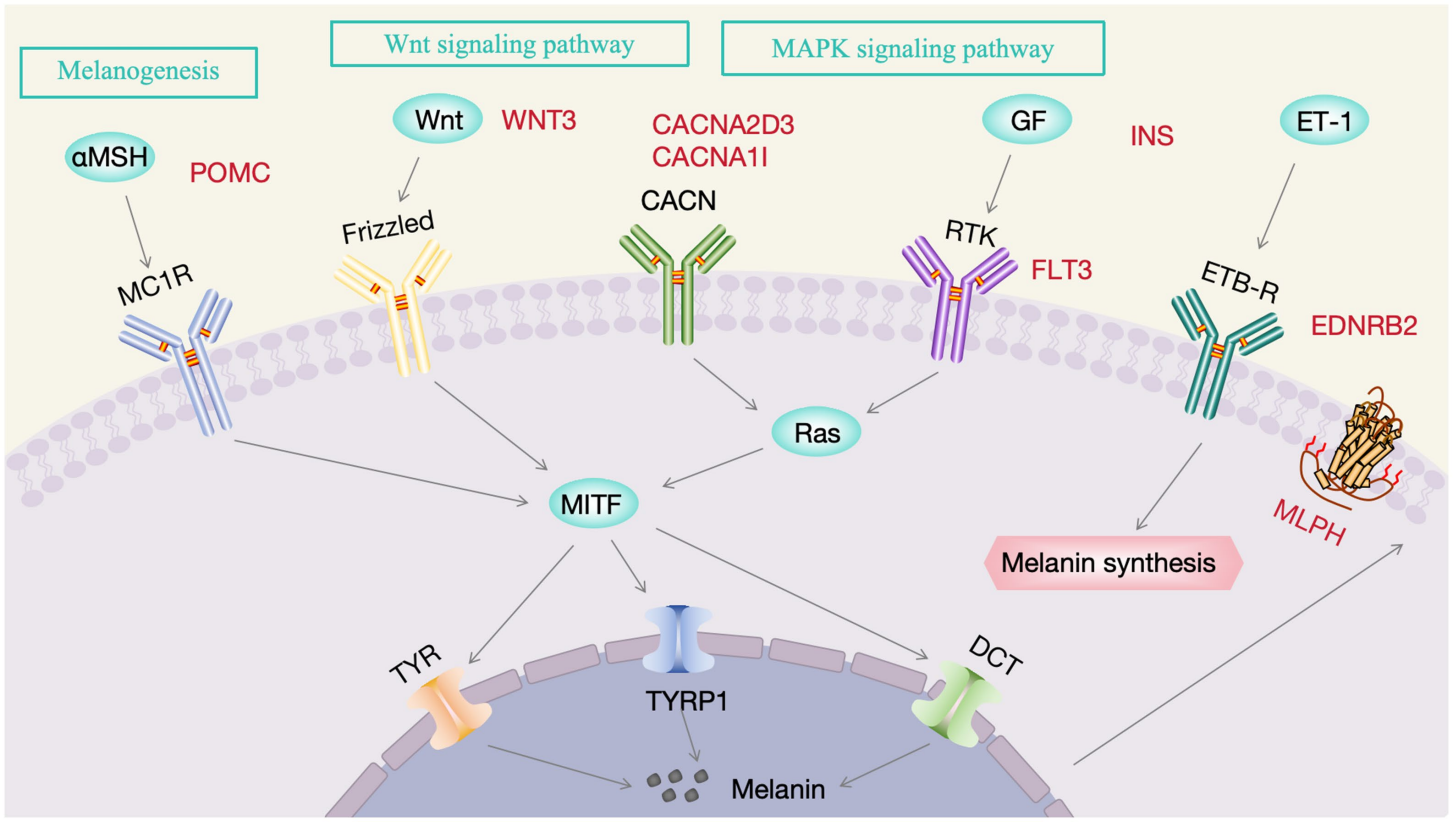
The mechanism of action of tyrosine on feather coloration. Red color indicates DEGs, while black represents non-DEGs.

As shown in Figure 6A, 72 DEGs were enriched in 5 KEGG Class A pathways and 16 KEGG Class B pathways. The KEGG enrichment circle plot revealed that the DEGs were the most enriched in neuroactive ligand–receptor interactions, the MAPK pathway and cytokine–cytokine receptor interactions (Figure 6B). Specifically, tyrosine supplementation resulted in significant changes in 4 pathways (*p* < 0.05), namely, “neuroactive ligand–receptor interactions,” “cytokine–cytokine receptor interactions,” “the intestinal immune network for IgA production,” and “valine, leucine and isoleucine biosynthesis” (Figure 6C). As indicated in Supplementary Table S4, the DEGs were associated with melanin-related pathways such as melanogenesis, the MAPK, Wnt, cAMP, PI3K-Akt, Notch, TGF-β, calcium, and mTOR pathways, as well as adrenergic signaling in cardiomyocytes. Notably, the network plot revealed relationships among the melanin-associated pathways involved in melanogenesis, MAPK and Wnt signaling; therefore, we focused on the 7 DEGs in these pathways (*EDNRB2, WNT3, POMC, INS, FLT3, CACNA2D3*, and *CACNA1I*; Figure 6D), which were likely to be involved in tyrosine-mediated feather color development (Figure 7).

### 3.7 DEG PPI network

**Figure 8:**
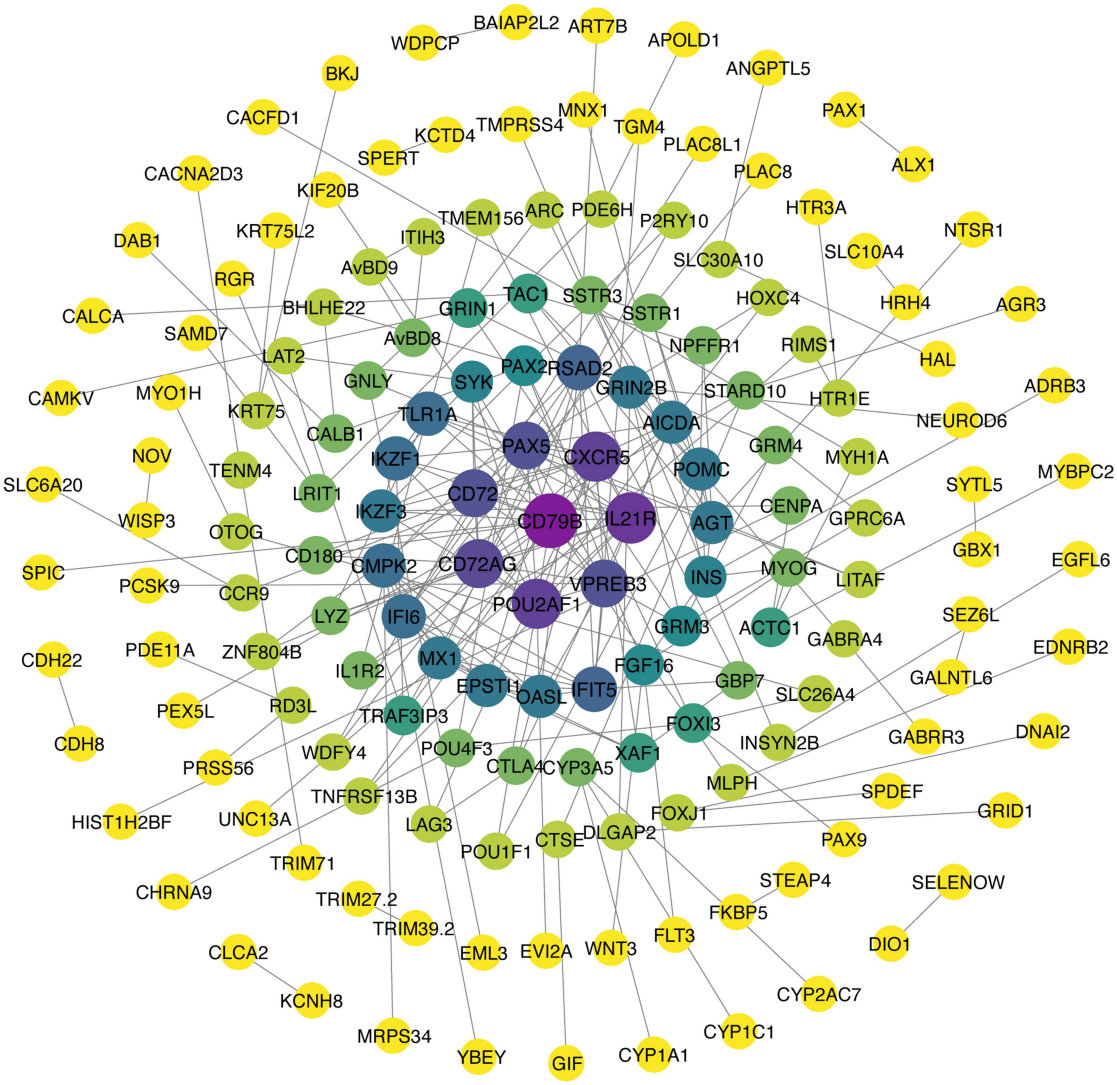
PPI network of DEGs. Nodes in the PPI network are colored on the basis of degree, with darker nodes indicating a greater degree.

The network consisted of 160 proteins and 172 interactions (Figure 8), among which the CD79B, IL21R, CXCR5, POU2AF1, CD72AG, CD72, PAX5, and VPREB3 proteins participated in more than 10 interactions. Notably, these networks are enriched in proteins involved in several melanin-related pathways. For example, POMC interacts with NPFFR1, INS, SSTR1, AGT, SSTR3, POU1F1, and TAC1, of which POMC participates in melanogenesis and INS participates in the MAPK and mTOR pathways. In addition, EDNRB2 interacted with MLPH, and WNT3 interacted with FGF16; among these, EDNRB2 and WNT3 showed enrichment in melanogenesis, whereas FGF16 showed enrichment in the MAPK pathway. Moreover, ACTC1, which is enriched in adrenergic signaling in cardiomyocytes, interacts with MYOG, CENPA, MYH1A, and MYBPC2; GRIN1, which is enriched in the calcium signaling pathway, interacts with GRIN2B, ARC, GRM3, and CAMKV. In addition, CACNA2D3 and FLT3, which are enriched in the MAPK signaling pathway, are connected to TENM4 and FGF16, respectively.

### 3.8 Temporal expression analysis of EDNRB2

**Figure 9:**
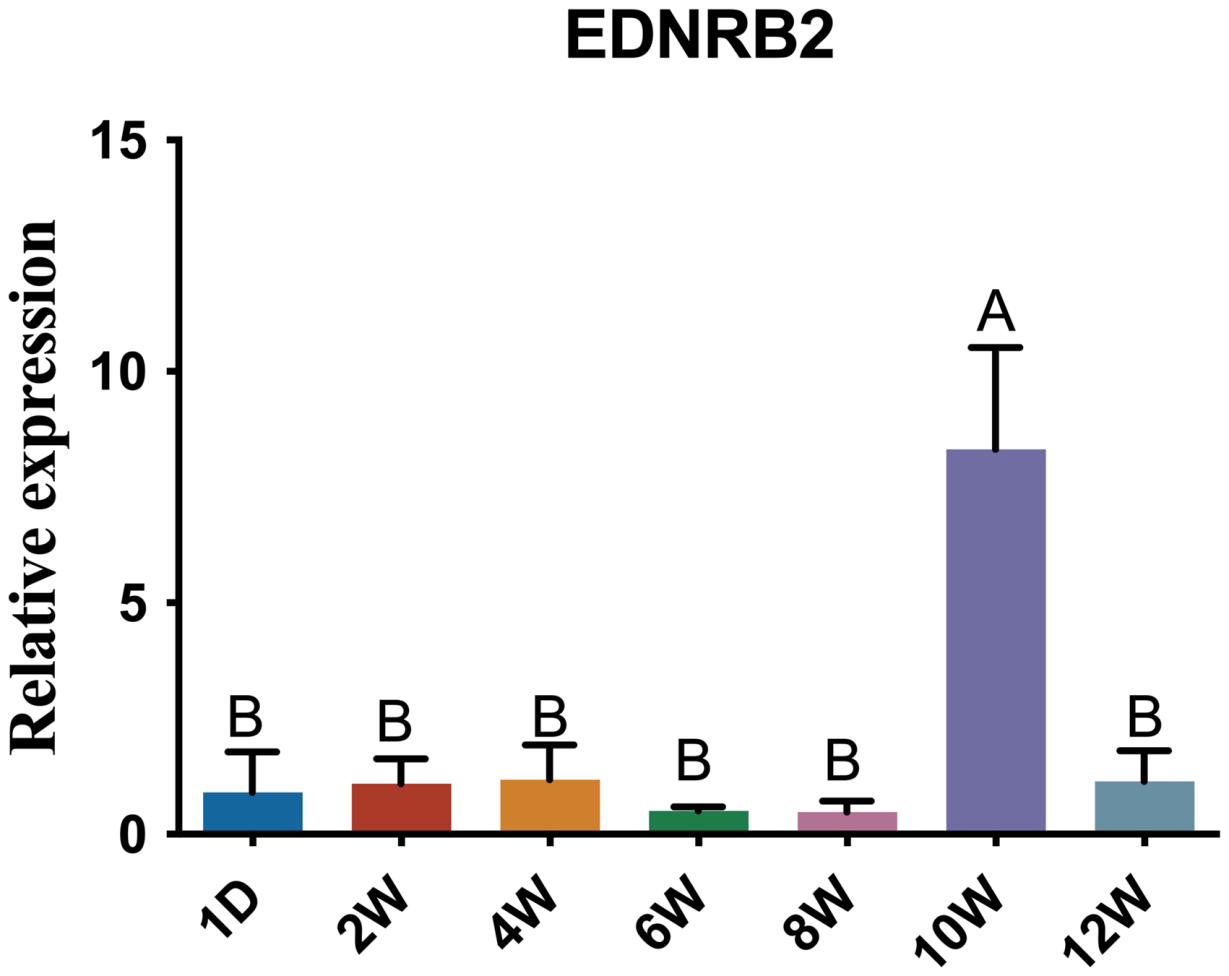
Expression profile of *EDNRB2* in the follicle tissue of H line hens at different stages. Data are expressed as mean ± SD (*n* = 6). Different uppercase letters within groups indicate highly significant differences (*p* < 0.01).

On the basis of the enrichment results of the KEGG pathways and PPIs related to the DEGs, we observed that *EDNRB2* is not only annotated in the melanogenesis pathway but also interacts with the melanosome transport protein MLPH. To investigate the temporal expression characteristics of *EDNRB2* at 1 d, 2 w, 4 w, 6 w, 8 w, 10 w, and 12 w in H line hens, we performed qRT–PCR with cDNA samples from neck follicle tissues at each time point (Figure 9). Notably, the expression levels of *EDNRB2* were the highest at 10 W, and the expression levels were significantly different from those at other time points (*p* < 0.01).

### 3.9 Analysis of EDNRB2 expression in chicken melanocytes after tyrosine supplementation

**Figure 10:**
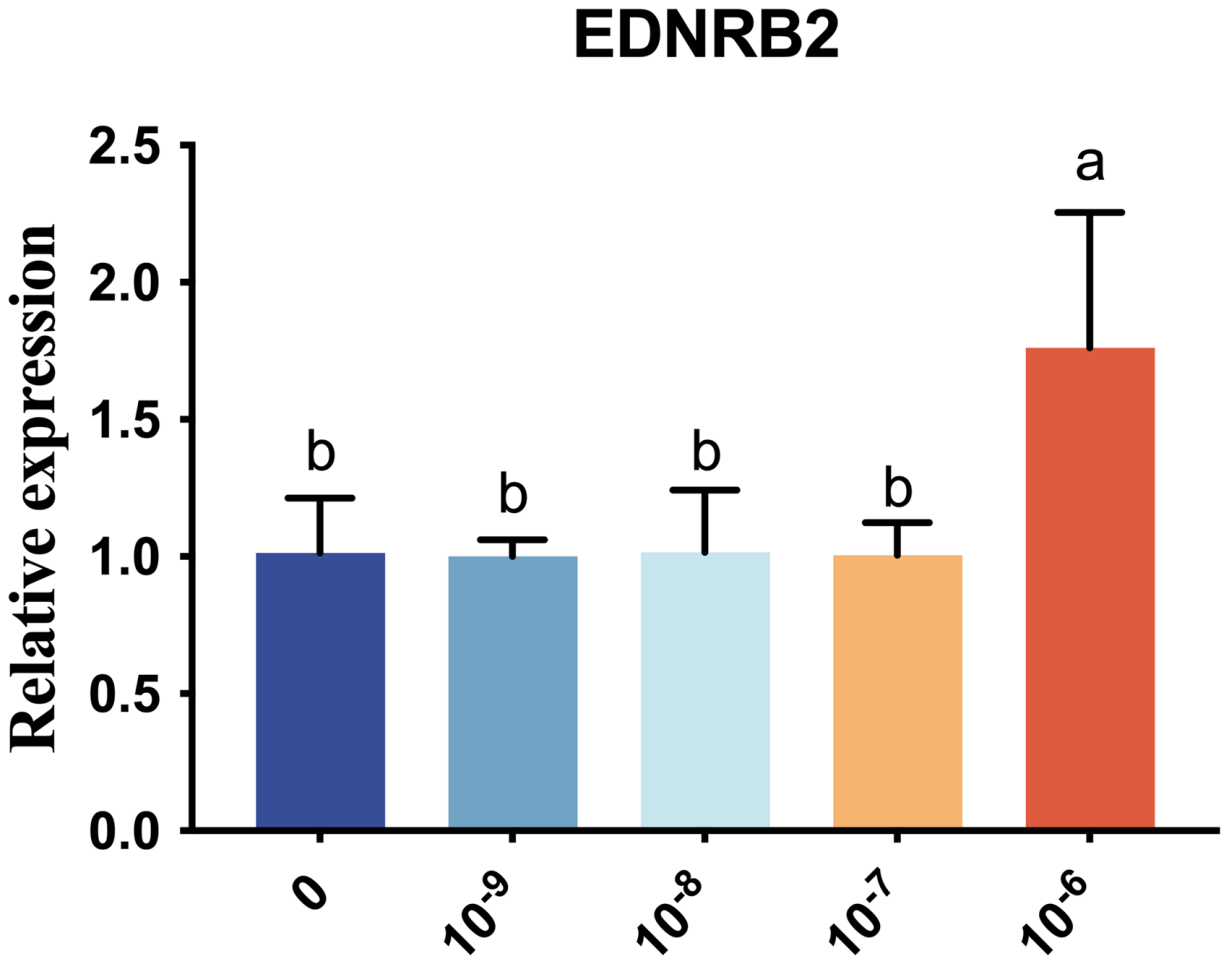
Expression profile of *EDNRB2* in primary melanocytes of chickens treated with different concentrations of tyrosine. Data are expressed as mean ± SD (*n* = 3). Different lowercase letters within groups indicate significant differences (*p* < 0.05).

In this study, chicken primary melanocytes were selected, and the expression of *EDNRB2* was verified by adding different concentrations of tyrosine to the cells. It was found that *EDNRB2* levels in chicken melanocytes were highest when the tyrosine concentration was 10^−6^ mol/L, and the differences were markedly greater than those of the control (0 mol/L) and 10^−9^, 10^−8^, and 10^−7^ mol/L treatment groups (*p* < 0.05) (Figure 10).

## 4 Discussion

The color of chicken feathers is influenced not only by genetic factors but also by nutritional factors. Although many of the genes related to feather colors have been characterized, the molecular mechanisms controlling melanin deposition in feathers in relation to dietary nutrient intake have not yet been fully explored. Tyrosine is known to be associated with melanin formation. Studies have shown that feeding tyrosine can increase the melanin content in animals, but there is no relevant research indicating that tyrosine can increase melanin in chicken feathers. In this study, we allocated chickens with sub-Columbian plumage to different tyrosine supplementation groups to determine the optimal dosage and feeding time for inducing black spots in feathers. The results indicated that dietary tyrosine affects the melanin content in feathers in a concentration-dependent manner. Specifically, when the tyrosine content in the feed is 1.0% and the feeding duration is 40 days, it can significantly increase the melanin content in the follicles (*p* < 0.05). Similarly, in previous work, dietary supplementation with 0.8% tyrosine was found to promote the deposition of melanin in the pectoral muscles of the Xichuan black-bone chicken (18). This study provides guidance for the nutritional regulation of feather color in the H line chickens and the breeding of excellent germplasm. These findings provide a theoretical foundation for the development and utilization of tyrosine in improving melanin deposition in chicken feathers.

Indeed, deciphering the effects of tyrosine-mediated gene upregulation or downregulation on feather follicle melanin deposition represents an interesting topic. The elucidation of the mechanisms responsible for melanin deposition in chicken feathers through tyrosine is extremely important for the development and utilization of sub-Columbian plumage. Transcriptome sequencing is a high-throughput method used to examine RNA molecules in tissues, with a focus on gene expression patterns and changes. Because black spots are present on the necks of chickens with sub-Columbian plumage, we selected a blank control group and an optimal feeding group of chicken neck follicle tissues for transcriptome sequencing to analyze the molecular mechanism through which tyrosine promotes melanin deposition in feathers. The marker gene *MLPH*, which is associated with melanin deposition, was detected in our transcriptome sequencing data. MLPH is a structural protein that facilitates the transport of mature melanosomes in melanocytes, which can cause melanosomes to aggregate in the dendrites of melanocytes and thereby regulate animal skin and coat color (31, 32). The expression of *MLPH* varies across tissues, as mature melanosomes are transported to dendritic terminals within melanocytes for short-distance movement, which is correlated with the expression levels and activity of the *Rab27a, MLPH*, and *MyoVa* ternary complex (33). Mutations in human *MLPH* can lead to Griscelli syndrome, which is characterized by abnormal skin pigmentation and light silver–white hair starting in infancy (34). The lavender diluted feather phenotype in chickens, akin to that of Japanese quail, is due to mutations in *MLPH* (35, 36). Combined with previous results, we hypothesized that the *MLPH* expression level is associated with tyrosine-mediated melanin deposition in the chicken.

Enrichment analyses were performed to elucidate DEG functions. KEGG pathway analysis revealed that the DEGs *EDNRB2, WNT3, POMC, INS, FLT3, CACNA2D3,* and *CACNA1I* were involved in melanogenesis and MAPK and Wnt signaling, all of which are known to be interconnected with each other. Melanogenesis is a complex biosynthetic process in melanocytes that leads to the production of melanin pigments through a series of enzymatic and chemically catalyzed reactions (37). *EDNRB2* and *POMC* both showed enrichment in the melanogenesis pathway, and their involvement in melanin production is well-documented. *POMC* is mainly secreted in the hypothalamus and acts as a precursor polypeptide hormone and is associated with the morphological differentiation and the production of melanin in B16-F10 melanoma cells (38, 39). Moreover, *POMC* is converted into hormones derived from it, including *ACTH* and *α-MSH* (39). *α-MSH* increases peroxide levels in melanocytes to stimulate the synthesis of melanin (40). *MITF* is a melanocyte-specific transcription factor that can directly bind and activate the tyrosinase promoter, upregulating tyrosinase levels and promoting melanin production (41). The Wnt axis is known to regulate *MITF* expression (42). This involves Wnt binding to the G-protein coupled receptor Frizzled, inactivating *GSK3*β and ultimately resulting in β-catenin accumulation (43–46). This β-catenin undergoes nuclear translocation where it binds to *Lef-TCF*, thereby increasing *MITF* expression and stimulating melanin synthesis (47, 48). *Wnt3* is part of a group of approximately 20 ligands involved in the Wnt axis, influencing cell proliferation and differentiation (49). Therefore, we speculate that *WNT3* may promote the proliferation of melanocytes through the WNT signaling pathway. In addition, an interaction between Wnt3 and FGF16 was found when comparing the C and T groups. FGF family proteins are typically involved in cell proliferation, differentiation, and survival, and it is thus suggested that an interaction between Wnt3 and FGF16 may promote proliferation (50). The MEK and ERK kinases associated with the MAPK axis are known to activate melanocyte receptors (51). Ligands bind to the extracellular domain of the receptor, activating a cascade (Ras–Raf–MEK–ERK) that leads to the upregulation of *MITF* (51). P38 phosphorylation induces *MITF* expression, in turin upregulating proteins associated with melanin production and thus melanin synthesis (52). The *INS* gene encodes insulin, which activates the insulin signaling pathway during the process of cell differentiation, thereby promoting cell maturation (53). *FLT3* is a type III receptor tyrosine kinase, and its ligand can induce dimerization and activate its intrinsic tyrosine kinase activity, leading to autophosphorylation and the initiation of several signaling cascades, including the MAPK signaling pathway (54, 55). *CACNA2D3* and *CACNA1I* are both gene families associated with calcium channels that play key roles in the regulation of intracellular calcium ion concentration, influencing cell proliferation and differentiation by affecting the MAPK signaling pathway (56). Thus, we speculate that these DEGs are involved in melanin production, although the precise genetic process requires further research.

Among the DEGs identified here, *EDNRB2* is worthy of attention because it not only participates in melanin-related pathways but also interacts with the MLPH protein. *EDNRB2*, an *EDNRB* paralog, encodes a seven-transmembrane-domain G protein-coupled receptor (26, 27, 57, 58). *EDNRB2* dysfunction affects pigment synthesis, leading to white fur and pink skin (59–61). *EDNRB2* is also associated with the migration and differentiation of melanocytes and has been linked to plumage color in quail, chickens, and domestic ducks (7, 27, 61, 62). Chicken feathers from the embryonic stage to adulthood undergo 3 ~ 4 generations of replacement, and we call this phenomenon production molting; after many molts, the feather color becomes stable. At 55 days of age, the third-generation feathers (young feathers) of chickens begin to appear, and the feather color on the body surface of chickens is mature or nearly mature and tends to be stable (63). Our own observations in the H-line chickens are consistent with this timeline: starting from yellow chick down, the chickens develop their characteristic sub-Columbian plumage pattern through successive molting, with the definitive coloration becoming stable by approximately 10 weeks of age. This developmental timeline explains the peak in *EDNRB2* expression at 10 weeks within the 0-12w expression profile, suggesting a crucial role for *EDNRB2* in the formation of the sub-Columbian plumage pattern and the associated melanin deposition process.

The black pigment in the feathers of chickens is produced in melanocytes through a series of biochemical reactions involving tyrosine, and many studies have indicated that adding tyrosine to melanocytes can promote melanin deposition (64). For example, the addition of tyrosine to culture medium promoted melanin synthesis in mouse B16 melanoma cells (65). Adding 400 μM L-tyrosine to Ham’s F-10 medium led to the induction of melanin pigment production in human melanoma cells (66). Treating *in vitro*-induced primary chicken melanocytes with varying amounts of tyrosine promoted melanin proliferation and increased the tyrosinase content in the cells (18). Here, marked increases in the levels of *EDNRB2* were observed after the addition of 10^−6^ mol/L tyrosine to chicken primary melanocytes. However, a previous study reported that the highest expression level of *EDNRB2* in chicken primary melanocytes was observed when 10^−9^ mol/L tyrosine was added, indicating that the optimal concentration for increasing *EDNRB2* may be related to the cell state and culture conditions (18). In summary, we confirmed that exogenously added tyrosine can promote the expression of *EDNRB2* at the cell level, thereby facilitating melanin deposition.

## 5 Conclusion

The results of our study reveal that a 40-day diet with 1.0% tyrosine can significantly intensify the darkness of black spots on the feathers of sub-Columbian (*p* < 0.05). To further explore the mechanism of action, we conducted transcriptomic sequencing analysis on the feather follicle tissues of the control group and the tyrosine treatment group (which received a diet supplemented with 1.0% tyrosine for 40 days) using RNA-seq technology. Tyrosine induction of melanin deposition in follicular tissues of chickens with sub-Columbian plumage might be significantly affected by the *EDNRB2*-mediated regulatory network. These results advance the knowledge of the molecular regulation of melanin deposition in sub-Columbian plumage, establishing a basis for feeding and breeding practices to modulate melanin levels in H-line chickens.

## Data Availability

The datasets supporting the conclusions of this article are included within the article and its additional files. Transcriptome sequencing data were deposited in the NCBI SRA database (SRA accession: PRJNA578584).

## Footnotes

1
http://deweylab.biostat.wisc.edu/rsem/rsem-calculate-expression.html

2
http://www.string-db.org
