# Supplementary material for: Transcriptomic insights into the effects of tyrosine on sub-Columbian plumage in H line chickens
Source: Front Vet Sci. 2025 Nov 28;12:1720520. doi: 10.3389/fvets.2025.1720520 (PMC12699944; doi:10.3389/fvets.2025.1720520)
Supplement: Supplementary file 1 [file Table_1.docx]

**Supplementary Table S1.** Composition and nutrient levels of the basal diets (air-dry basis).

| Ingredients | Content (%) | Nutrition level | Content(%) |
| --- | --- | --- | --- |
| Corn | 65.6 | ME (MJ/kg） | 17.5 |
| Soybean meal | 20.7 | CP (%） | 16.02 |
| Wheat bran | 9.82 | Ca (%） | 0.79 |
| Limestone | 1.20 | AP (%） | 0.35 |
| NaCl | 0.30 | Met (%） | 0.3 |
| Ca HPO4 | 1.30 | Lys (%） | 0.74 |
| Premix | 1.00 |  |  |
| Total | 100.00 |  |  |

Abbreviations: The premix provided per kg of diet: VA 8000 IU, VD3 2800 IU, VE 20 mg, VK3 2 mg, VB 9.62 mg, Mgnicotinic acid amide 32 mg, D-calcium pantothenate 9 mg, Folic acid 1.1 mg, Biotin 0.12 mg, Fe 64 mg, Mn 96 mg, Zn 64 mg, Cu 8 mg, I 0.6 mg.

**Supplementary Table S2.** qRT-PCR primer sequences.

| Gene | Sequence (5’ to 3’) | Product size（bp） |
| --- | --- | --- |
| *WNT11* | F: AGGTGAGACACCTGGACCTG  R: GGAGACAGGTCTTGAAAGCCTC | 180 |
| *WNT3* | F: CCGGGAGGACGACCATTCT  R: ACCATCTCGGAAGCACTGTC | 157 |
| *GNAQ* | F: CCTGAGCGAGGAAGCCAAG  R: CTCTCTCCTGTACCGAGCAC | 140 |
| *PVALB* | F: AGGAGAGATCCGAAAGTTGC  R: AAGCCGCTCCGGTCTTTATC | 196 |
| *EDNRB2* | F: CTTCATGGTGGCCATTCTGG  R: GGTCTTGGTCTGATCGTGCC | 105 |
| *PRKCB* | F: GCAAAGGGCTCATGACCAAG  R: TTCAGCATTGCGTCCACAAG | 167 |
| *GADPH* | F: TGATGGTCCACATGGCATCC  R: GGGAACAGAACTGGCCTCTC | 141 |

**Supplementary Table S3.** Statistics of quality control and reads mapping of sequencing date.

| Sample Name | Raw Reads/ Mb | Clean Read/ Mb | Q20 (%) | Q30 (%) | Clean Read Ratio (%) | total mapping  (%) | uniquely mapping  (%) |
| --- | --- | --- | --- | --- | --- | --- | --- |
| C1 | 52.59 | 43.67 | 96.71 | 88.47 | 83.05 | 82.40 | 55.95 |
| C2 | 50.83 | 43.40 | 96.31 | 87.19 | 85.37 | 85.36 | 56.39 |
| C3 | 50.83 | 44.07 | 96.32 | 87.24 | 86.70 | 86.95 | 57.64 |
| T1 | 50.83 | 43.04 | 96.62 | 88.23 | 84.66 | 86.42 | 58.30 |
| T2 | 50.83 | 43.55 | 96.34 | 87.31 | 85.68 | 86.39 | 57.04 |
| T3 | 50.83 | 43.71 | 96.53 | 87.89 | 85.98 | 86.39 | 57.69 |
